# Understanding Elderly Drivers’ Perception of Advanced Driver Assistance Systems: A Systematic Review of Perceived Risks, Trust, Ease of Use, and Usefulness

**DOI:** 10.3390/geriatrics9060144

**Published:** 2024-11-05

**Authors:** Federica Biassoni, Martina Gnerre

**Affiliations:** Traffic Psychology Research Unit, Department of Psychology, Catholic University of the Sacred Heart, Largo Gemelli 1, 20123 Milan, Italy

**Keywords:** ADAS, older drivers, traffic psychology, risk perception

## Abstract

Background: Elderly drivers often face safety challenges due to age-related declines in cognitive, sensory, and motor functions. Advanced Driver Assistance Systems (ADAS) offer a potential solution by enhancing safety and mobility. Objectives and method: This systematic review investigates the factors influencing the perception and usage of ADAS among elderly drivers, focusing on perceived safety, usefulness, trust, and ease of use. Results: Older adults show a preference for Level 1 ADAS, which they perceive as safer. Although they acknowledge the usefulness of ADAS in supporting their autonomy, skepticism remains regarding higher-level systems, primarily due to concerns about reliability and invasiveness. Trust and ease of use are essential factors influencing their acceptance. The review identifies common themes and barriers to the adoption of these technologies and emphasizes the need for senior-friendly interfaces and targeted training. The findings indicate that addressing these issues can significantly improve the safety and mobility of elderly drivers. The successful adoption of ADAS among older adults depends on balancing safety, control, and ease of use, with gradual and supportive integration fostering greater acceptance and trust. Conclusions: This study outlines practical implications for stakeholders, emphasizing the need for user-friendly ADAS design, public awareness campaigns, government incentives, insurance discounts, and community training to enhance adoption among older drivers.

## 1. Introduction

As the global population ages, the number of elderly drivers is increasing, raising the issues of their unique needs and challenges on the road [1,2]. Elderly drivers often face age-related declines in cognitive, sensory, and motor functions, which can impact their driving performance and safety [3].

Advanced in-vehicle technologies, such as Advanced Driver Assistance Systems (ADAS), can help mitigate these declines, promoting safer driving practices and increasing mobility [4,5].

Recent studies have demonstrated that assistance systems can significantly reduce the occurrence of on-road accidents [6,7,8].

Technologies such as adaptive cruise control, lane departure warnings, automatic emergency braking, and parking assistance are designed to support drivers in various tasks [9,10].

However, older drivers often do not use ADAS effectively. A study by the AAA Foundation for Traffic Safety found that many elderly drivers either figured out ADAS functionalities by themselves or did not learn how to use them, leading to underutilization of these technologies [11]. Despite the increasing availability of ADAS features, older drivers’ usage rates have remained relatively low, with many reporting rare or no use of these systems [12].

The adoption and effectiveness of ADAS among elderly drivers could be influenced by their perception of risk, perceived usefulness, perceived ease of use, and trust in these technologies [10]. Understanding how the elderly perceive the risks and benefits of ADAS is crucial for the design, implementation, and hence acceptance of such systems.

This systematic review aims to explore the existing literature on the factors related to ADAS acceptance among the elderly. By synthesizing findings from various studies, we seek to identify common themes, factors influencing acceptance (such as risk perception, trust, ease of use, usefulness), and potential facilitators and barriers to the adoption of ADAS by elderly drivers.

### 1.1. Safety Perception

Safety perception reflects the degree to which a driver believes that using an ADAS system is safe [13,14]. Although ADAS are designed to increase safety, concerns persist regarding potential driver over-reliance and a subsequent reduction in vigilance [14]. In some cases, drivers’ risk perception may compensate for the ADAS benefits, where riskier behaviors are adopted due to a false sense of heightened protection [15]. In relation to older drivers, cognitive, sensory, and motor declines associated with aging can affect how they perceive the risks associated with new technologies, in one way or another [16]. For example, these changes can lead to a negative perception of technologies if they are poorly designed for the capabilities of older adults [16], or, conversely, if well designed, they may result in increased use of technologies [17].

### 1.2. Trust

Trust is defined as the willingness to rely on another party based on their qualities [18]. Older individuals generally trust automation more than younger people, though this can vary depending on the situation [19,20]. Experiential knowledge and prior usage can enhance a driver’s understanding and trust in automation [21,22]. Despite the safety benefits of ADAS, older drivers often show increased caution, affecting their trust in these technologies [23]. Factors influencing technology use in vehicles include perceived needs, cognitive abilities, prior experience, and attitudes [16]. Older adults tend to be more resistant to new technologies due to the difficulty of learning new skills and changing routines with age [24,25,26].

### 1.3. Perceived Usefulness and Perceived Ease of Use

The Technology Acceptance Model (TAM) has received extensive theoretical and empirical validation [3,27,28]. This model suggests that a user’s attitude toward and intention to use a system are primarily influenced by two factors: the perceived usefulness of the system in enhancing performance and the perceived ease of use. Perceived usefulness is defined as the extent to which an individual believes that employing a specific system will improve their job performance [27]. Perceived ease of use is the degree to which a person believes that using the system will require minimal effort [28]. Numerous studies have applied this model to evaluate driver acceptance of ADAS technology and confirmed the influence of the two factors [29,30].

## 2. Review Methodology

This systematic review adheres to the Preferred Reporting Items for Systematic Reviews and Meta-Analyses (PRISMA) guidelines. As this research involved reviewing previously published studies, no ethical approval was necessary. The following section details the procedures used to identify, select, and analyze the relevant literature on the perception of risk in ADAS among older drivers.

### 2.1. Inclusion and Exclusion Criteria

To determine eligibility, specific criteria were established. We included studies involving elderly individuals aged 60 and above that focused on various types of ADAS. The standard for levels of automation was established by SAE International in 2014, outlining a scale for autonomous driving ranging from fully manual (Level 0) to fully automated (Level 5) (Table 1). In this research, the interest is only from 1 to 3. Both qualitative and quantitative studies were considered, encompassing surveys, interviews, observational studies, and experimental designs. Only studies published in English from 2015 to 2024 were included. The focus was exclusively on cars, with other vehicles such as trucks and trains excluded. Systematic reviews, meta-analyses, essays, master’s or doctoral theses, and non-peer-reviewed papers were excluded. We did not include studies involving elderly individuals with dementia or other debilitating conditions. Instead, we focused on studies with participants aged 60 and older.

### 2.2. Search Strategy

Between May 2024 and July 2024, we conducted a comprehensive search across several electronic databases: PubMed, Scopus, Science Direct, EEE Xplore, TRID, and PsycINFO. Additionally, we manually searched the reference lists of relevant articles to identify any additional studies. The search terms included: (“Advanced Driver Assistance Systems” OR “ADAS”) AND (“elderly” OR “older adults” OR “older drivers”) AND (“perception of risk” OR “risk perception” OR “perception of safety” OR “perceived risk” OR “hazard perception” OR “safety perception” OR” feelings of safety” OR “perceived safety”) OR (“perceived utility” OR “perception of utility” OR “perceived usefulness” OR “perception of usefulness”) OR (“trust”). We conducted searches across the same databases using identical keywords.

### 2.3. Data Extraction

In this systematic review, data extraction and study quality assessment were meticulously conducted to ensure the reliability and validity of the findings. Initially, all identified records were imported into Rayyan software, which facilitated the removal of duplicate entries and the initial screening. After initially screening the titles and abstracts, the two authors reviewed the full texts to make selections. Eligible studies were then carefully selected based on a thorough review of their full texts.

The data extracted from these studies included key details such as author(s), year of publication, country, study design, type of ADAS investigated, SAE automation level, the main constructs examined, the experimental design, and the main results. We listed and defined all sought outcomes, including risk perception, trust, perceived usefulness, and ease of use, and ensured all compatible results were collected from each study.

### 2.4. Quality Assessment

To assess the risk of bias, we utilized the standardized QualSyst evaluation tool [31]. The assessment of the methodological quality was conducted after the data extraction process for each record that was not excluded during the title and abstract review. Two authors independently conducted the quality assessment of the studies. This framework includes distinct scoring systems for qualitative and quantitative research. The qualitative section includes ten items, each rated on a scale from 0 to 2, with a maximum possible overall quality score of 20. The quantitative scale consists of 14 items, also scored from 0 to 2, with a maximum total quality score of 28. Summary quality scores (SQSs) were presented as percentages of the total possible score, ranging from 0 to 100%, with a higher SQS reflecting greater methodological quality. No studies were excluded based on methodological quality. The reviewed articles generally had clearly defined research objectives, appropriate study designs, and a well-articulated context, connecting the studies to a broader theoretical framework or body of knowledge. In all the selected articles, the subject selection methods were adequately described and deemed appropriate. The sampling strategies were relevant and justified, and the characteristics of the subjects were clearly detailed. Although two studies did not fully detail their selection methods, no major concerns were identified. All articles reported their results in sufficient detail, and none of the quantitative studies suffered from inadequate sample sizes. Additionally, the conclusions drawn in all reviewed articles were well-supported by the results. In qualitative studies, verification procedures to establish credibility were consistently mentioned. Notably, one study did not specify the type of ADAS investigated, but we included it as it still aligned with our objectives.

### 2.5. Study Selection

**Figure 1:**
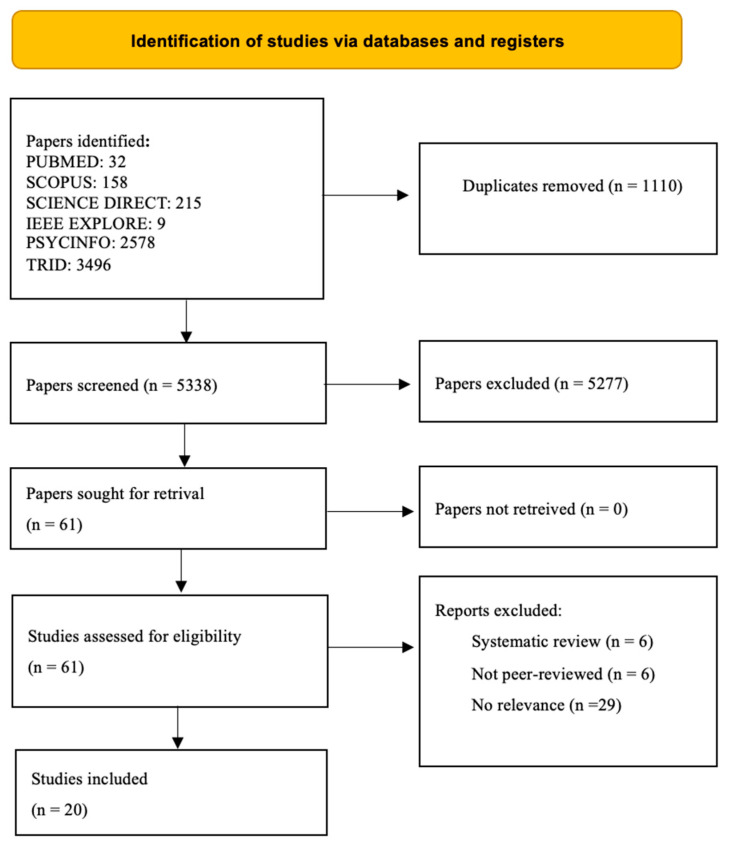
PRISMA flow diagram for study selection.

The studies were screened by full text, which in turn led to 61 studies being selected for full-text screening. A total of 20 studies were funded (Figure 1). After selecting the studies to be included, the following data were extracted: initial author and year of publication; country in which the study was conducted; keywords; aim of the study; study design and measures used; sample size as well as age and gender of participants; outcomes and measurements. The results from the selected studies are summarized below.

## 3. Results

### 3.1. Methodological Overview of the Studies

A total of 20 papers were included in the systematic review. We organized the study results using a structured table (Table 2), which was compiled manually. In the table, we included the following variables: First Authors, Year, Country, Methods, Level of ADAS, Construct, and Main results, to organize and present the key information from each study systematically. The highest number of studies were published in 2018 (six studies), followed by 2021 (five studies). These two years represent the periods with the most publications among those listed. The highest number of studies were conducted in the USA (seven studies), followed by Japan (five studies) and Germany (four studies). These three countries represent the majority of the included studies. The studies utilized a variety of research methods to gather comprehensive insights. Surveys were a common approach, employed in multiple studies [32,33,34,35,36] to collect quantitative data on users’ attitudes, acceptance levels, and perceived usefulness. Experimental studies, often involving driving simulators, were frequently used to assess participants’ trust and perceived usefulness of ADAS in controlled environments [37,38,39,40,41,42,43,44]. These methods allowed for detailed observations of interactions with ADAS in simulated driving conditions. Interviews and focus groups provided qualitative insights into older adults’ perceptions, concerns, and preferences, allowing for a deeper understanding of the nuanced factors influencing ADAS acceptance [45,46,47,48]. Some studies employed mixed methods, integrating surveys, interviews, and experimental approaches to triangulate data and validate findings across different methodologies [23,49]. This multi-faceted approach enabled researchers to capture a holistic view of older adults’ interactions with and attitudes towards ADAS technologies. Saito et al. [50] is the only study that incorporated telemetric data alongside real-world driving experiments to objectively measure the impact of ADAS on driving performance and safety perceptions.

### 3.2. Level of ADAS

This section explores older adults’ preferences regarding different levels of ADAS. Older adults show a clear preference for Level 1 ADAS systems, such as Lane Keeping Systems (LKSs) and Adaptive Cruise Control (ACC), due to their ability to enhance driving control and safety and also to reduce fatigue on long drives [23,32,36,49]. The actual use of systems like Lane Keeping Assist (LKA) is s not widely accepted, as indicated by three studies [23,32,37]. Also, proactive steering intervention is not really accepted [41,42,49]. Gish et al. [46] found that older drivers tend to perceive the Lane Departure Warning system positively. However, Eichelberger and McCartt [37] noted that, in comparison to other crash avoidance systems, lane departure warning and prevention features are consistently reported as the least used by vehicle owners. Backup cameras and Blind Spot Monitoring (BSM) are particularly valued for improving safety during parking and lane changes, respectively [32,36,46]. Some participants specifically sought technologies like blind spot monitoring and backup cameras due to visual impairments [46]. Xu et al. [36] conducted a survey involving healthy elderly individuals and elderly individuals with vision impairments. The study showed that, unlike drivers with vision impairments, healthy elderly participants were less inclined to use active collision prevention systems and were more open to technologies that provide information without active intervention. There is a general distrust of ABSs among older adults, especially older women, due to concerns about invasiveness and reliability [32].

However, systems that enhance situational awareness and perception are well-accepted. Blind Spot Monitoring (BSM), for example, is positively evaluated as it helps prevent accidents caused by blind spots, a major concern during lane changes [23,32].The Proactive Braking Intervention was also more accepted than the Proactive Steering Intervention [41,42,43]. Such a higher level of acceptance is likely due to the more intuitive nature of braking as a safety response compared to steering, which can be more complex and less predictable for elderly drivers. Moreover, older adults prefer systems offering information or warnings over fully automated features [49]. Research indicates high acceptance rates for ADAS among older adults, with many preferring features that reduce driving stress and improve comfort [23,32,36,49].

Overall, the adoption and use of ADAS among older adults are influenced by perceived risk, trust, and the usefulness of the systems. Level 1 and 2 systems are generally preferred for their supportive roles without fully automating driving tasks, while Level 3 systems require further development to build trust and acceptance [49]. While older adults recognize the utility of Level 3 systems for enhancing autonomy, especially in complex urban environments, they prefer to maintain some control over the vehicle for confidence’s sake [38,50].

### 3.3. Perceived Usefulness

Perceived usefulness is a critical factor influencing the acceptance of ADAS among older adults [33,34,35,37,38,40,45,46,49]. Older drivers found ADAS to be useful in counteracting age-related changes in driving performance [46]. Picco et al., [35] found that perceived usefulness tended to increase with age. Older drivers perceive ADAS that support speed regulation as more useful than devices relying on partial vehicle automation [49]. In particular, older women tend to evaluate ADAS as more useful compared to men; however, as the level of automation increases, the perceived usefulness becomes similar for both genders [49]. Günthner et al. [40] demonstrated that when older adults perceive these systems as beneficial and trustworthy, they are more likely to accept and use them. They recognize the usefulness of ADAS for increasing driving comfort and reducing stress, particularly appreciating features like Back-Up Cameras (BUCs) and Parking Sensors (PSs) for everyday tasks [45]. However, there is some skepticism about the necessity of more advanced systems, such as proactive steering interventions [37].

Older adults primarily attribute the usefulness of ADAS to maintaining mobility and autonomy despite health issues. They value the ability to relax while driving and having a system that supports their mobility needs [38].

Additionally, Bellet et al. [49] found that perceived utility is greater among men than women. Moreover, a study suggested that elderly individuals who perceive a high level of social cohesion in their neighborhood and have a good understanding of how the systems work tend to view ADAS as more useful [33]. In contrast, another study found that some elderly individuals consider ADAS unnecessary, believing they do not need them.

### 3.4. Safety Perception

Older adults’ perceptions of safety and their preferences for specific types of ADAS also play a crucial role in their acceptance of these technologies [34,46,50]. Asmussen et al. [32] highlighted that older adults are more likely to adopt ADAS that enhance driving control and safety, such as Lane Keeping Systems (LKSs), Backup Cameras (BUCs), and Adaptive Cruise Control (ACC). Also, Gish et al. [46] showed that technologies such as lane-departure warnings and adaptive cruise control were seen as enhancing safety by providing additional support and compensating for slower reaction times and decreased physical abilities. However, they are less likely to adopt Automatic Braking Systems (ABSs) due to concerns about its invasiveness and reliability [32,37,45]. Bellet et al. [49] noted that while older adults generally have a positive view of ADAS and are open to adopting new technologies, they prefer systems that provide information or warnings rather than those that fully automate driving functions. This preference suggests that older adults value maintaining a degree of control over their vehicles [37,50]. For example, they tend to rely less on automatic systems and more on manual intervention [50]. The results showed that more than half of the elderly participants applied the brakes themselves, even after the system initiated braking, indicating a desire for control and additional safety confirmations [50]. Proactive braking intervention significantly improves safety by reducing vehicle speed at critical points and increasing drivers’ awareness of potential hazards, especially in blind spots [50]. However, there is a general lack of understanding of how these systems work, leading to a desire for reassurance rather than technical details [37,45].

### 3.5. Trust

Trust in ADAS plays a crucial role in determining older adults’ willingness to adopt these technologies [41,42,43]. Older women in particular tend to have lower trust in highly automated systems, such as Automatic Braking Systems (ABSs) [32], a finding not supported by Zahabi et al. [44].Trust in automated systems is closely linked to perceived safety [23,32,42,43]. Positive impressions about safety improvements are mixed with concerns over false alerts and limited effective operation ranges [23]. For example, false alerts were a notable concern, affecting trust in ADAS [23]. Older adults generally show greater trust in systems that enhance situational awareness and reduce workload without removing vehicle control. Trust increases with experience and familiarity with the systems. Older adults who have previously used ADAS tend to have more trust in new systems compared to those who have never used them [32]. Liang et al. [23] found that older drivers recognize the safety benefits of ADAS systems, which can help build trust over time. Xu et al. [36] reported that participants perceived potential benefits of ADAS technologies to support their driving, indicating a general willingness among older adults to consider using ADAS if perceived as useful and beneficial.

Trust in the proactive braking intervention system was relatively high [41,42,43]. Participants expressed concerns about system failures or malfunctions, indicating that reliability is crucial for building trust [41,42,43,48] described a phased process of learning to use rear view cameras by becoming acquainted with the technology and testing its limits, demonstrating how older drivers engage in trust-building activities. Trust in ADAS needs to be developed over time through use and positive experiences with the technology [38]. Automated driving experience increased self-reported trust in automation among participants [39]. Older participants showed greater trust in automation compared to younger participants (under 30 years). They also rated the vehicle automation system more positively and expressed a greater intention to use such systems in the [39].This increase in trust was measured through questionnaires administered before and after the driving simulation experiment [39].To build trust, it is essential for users to directly experience the benefits of assistance systems [40]. For the 60–69 age group, perceived usefulness and trust in technology are more significant, while for the 70–90 age group, trust in technology is fundamental [40].

### 3.6. Ease of Use

The perceived ease of use of ADAS significantly influences their adoption among older adults. Simpler systems that require minimal learning are preferred, as older adults are more likely to use and trust ADAS that they find intuitive and easy to operate [37,45,46]. Comfort with this technology is associated with convenience, ease of use, and increased feelings of safety [46].

Moreover, a study suggested that elderly individuals who perceive a high level of social cohesion in their neighborhood and those who understand how the system functions tend to view ADAS as easier to use [33].Moreover, Kamide et al. [33] found that gender did not significantly influence the ease of use of the system. Another study [34] indicated that, compared to risk perception and perceived usefulness, ease of use has a more limited impact. This is likely due to the challenge of accurately measuring this factor without allowing participants to directly experience the technology.

### 3.7. Barriers and Facilities

Various existing barriers to the acceptance and use of ADAS emerged. Firstly, barriers to the adoption of safety technologies among older adults include a lack of knowledge and understanding of these technologies, the perception that non-standard features come with additional costs, and concerns about the expenses related to repair and maintenance of safety technologies [23,33,42,45]. The high cost of ADAS is another significant barrier, as these technologies are often available only as optional features on luxury vehicles or as expensive add-on packages, limiting their adoption by a broader segment of drivers [37].

Many drivers have also reported confusion or misinterpreting the mode of operation of ADAS, which can lead to cognitive overload, thereby reducing the system’s effectiveness and acceptability [37,43]. Moreover, older drivers expressed concerns about the rapid pace of technological advancements, finding it challenging to keep up with and understand these changes [38]. Additionally, there is a general lack of information and awareness about these systems, with many users unaware of their existence and functionalities [23,38]. For example, in the study by Günthner et al. [40] only 66% of users were familiar with distance control systems, and only about half knew of systems like Lane Departure Warning or Blind Spot Assistance. Some interventions, particularly steering, were perceived as momentarily stressful and intrusive [41,42].

Moreover, some elderly drivers considered proactive collision avoidance systems unnecessary because they trusted their own driving skills [42]. Despite these challenges, many older adults expressed interest in using ADAS in the future, especially as they age or if they experience cognitive or physical impairments [49]. The study also revealed that a significant portion of participants, particularly men, currently use and are highly interested in innovative in-car systems [49]. Intuitive and less overwhelming visual content can increase trust and, consequently, the acceptance of ADAS [49]. Clear and understandable visual information about the system’s actions improves acceptability and trust among elderly drivers [42,43]. Increased exposure and experience with ADAS could potentially enhance trust, as familiarity with the technology helps improve users’ mental models and attitudes toward it [44]. Moreover, a study suggests that social cohesion and understanding of system functionalities are key factors in promoting interest and acceptance of advanced driver assistance technologies among the elderly ([33]. Interestingly, many older adults also report that using ADAS reduces stress [23,32,37,47].

As a matter of fact, this review highlighted that there is a need for specific training programs for elderly drivers [23,34,40,44,46,48].

Accordingly, acceptance and proper use of ADAS may be supported through intuitive, senior-friendly user interfaces, in-depth training programs, and owner’s manuals specifically designed and tested for senior drivers [23,34,47].

## 4. Discussion

The reviewed studies provide a comprehensive overview of the factors influencing older adults’ perceptions of ADAS. Contrary to many age-related stereotypes about technology use, this study shows that the acceptance of ADAS tends to be high. A key finding is the general preference for Level 1 ADAS systems, such as Lane Keeping Systems (LKSs) and Adaptive Cruise Control (ACC), due to their supportive roles in enhancing driving safety and control [23,32,36,49]. These systems are preferred for providing information or warnings rather than fully automating the driving process, allowing older drivers to maintain control and thus feel more secure [40]. This preference aligns with the general mistrust observed towards more invasive technologies, such as Automatic Braking Systems (ABSs), which some older adults view as unreliable or overly intrusive [32,37,45]. Furthermore, as the use of fully automated vehicles continues to increase in the future, these factors could influence their adoption among older adults.

Older adults appreciate ADAS features that help compensate for age-related declines in driving capabilities, such as Back-Up Cameras (BUCs) and Blind Spot Monitoring (BSM) [32,36,45,46]. These systems are seen as particularly valuable for managing common challenges due to the normal decline of physical and perceptual functions that comes with age, like parking and lane changes. In sum, older adults are concerned about safety and seek systems that empower them rather than replace their driving capabilities. This need for empowerment is significant, as it underscores the psychological aspect of driving among older adults, who may already grapple with feelings of vulnerability due to age-related changes in physical abilities.

The perceived usefulness of these systems tends to increase with age, suggesting that as physical abilities decline, the appreciation for supportive technologies grows [33,34,37,38,40,45,46,49]. However, there is notable skepticism towards systems that involve proactive interventions, such as proactive steering, which may be perceived as reducing the driver’s sense of control. Older adults generally exhibit higher trust in systems that enhance situational awareness without taking over control [32,36,38,41,42,43]. Trust is often built through familiarity and positive experiences with these technologies [44].

For instance, older drivers who have previously used simpler ADAS tend to trust newer technologies more.

Conversely, a lack of understanding or experience with these systems can lead to distrust, particularly if there are concerns about system failures or false alerts. This distrust is more pronounced in complex or highly automated systems, where the technology’s actions may not be fully understood by the user. The ease of use is also a critical determinant of ADAS adoption among older adults [37,45,46]. Systems that are intuitive and straightforward are more likely to be accepted and used regularly. Complex interfaces or functionalities can lead to confusion, frustration, and ultimately rejection of the technology.

Integrating the interconnected themes of trust, ease of use, perceived usefulness, and perceived safety clearly demonstrates that successful ADAS adoption among older drivers requires a multi-faceted approach, where each factor plays a crucial role. Trust is fundamental, as older adults are more likely to adopt systems that they feel confident in. Perceived usefulness promotes adoption when ADAS effectively addresses age-related challenges, while minimizing perceived risks—such as loss of control—ensures that older drivers feel comfortable and secure using the technology.

Several key limitations were identified. Despite utilizing comprehensive search strategies across multiple databases, there remains a possibility that relevant studies were missed, which could influence our findings. This limitation arises from factors such as the limited number of databases searched, differences in terminology across various research fields, and the potential existence of grey literature or ongoing studies that were inaccessible during our search. Additionally, we only reviewed studies published in English, which may have led to the exclusion of some relevant international research. Moreover, a notable limitation is that only three studies have examined and discussed the gender variable in relation to age. This restricts our ability to fully understand how gender differences may impact the perception and use of ADAS among older drivers. Future research is needed to explore further the role of gender in ADAS adoption and perception.

### 4.1. Practical Implications

This study presents some practical implication to meet the specific needs of different stakeholders. For technology developers, the design of intuitive, user-friendly ADAS interfaces should be prioritized. Simpler, more accessible systems that provide clear and concise information without overwhelming users are more likely to be adopted by older drivers. Governments can play a vital role in promoting ADAS adoption by offering incentives such as subsidies or tax reductions, making these systems more affordable for elderly drivers. Additionally, regulations mandating the inclusion of essential ADAS features in all new vehicles would help enhance road safety across all age groups. Public awareness campaigns highlighting the safety benefits of ADAS could further encourage adoption. Furthermore, policymakers should consider strategies to lower the cost of ADAS technologies, such as offering these systems as standard features rather than as expensive add-ons. For insurance companies, offering discounts to older drivers who use ADAS-equipped vehicles can provide a strong financial incentive for adoption. This approach not only helps reduce costs for drivers but also aligns with the broader goal of decreasing accident rates, ultimately benefiting both insurance companies and drivers. Finally, local communities can provide free or low-cost ADAS training sessions. These programs can empower older adults to confidently adopt and use these technologies. Comprehensive educational efforts, including workshops, instructional materials, and hands-on demonstrations, would significantly improve their understanding and comfort with ADAS, fostering familiarity and trust. By engaging these stakeholders with specific actions, the adoption and effective use of ADAS among elderly drivers can be enhanced, ultimately improving road safety and mobility for this growing demographic.

### 4.2. Conclusions

In conclusion, while older adults show a cautious acceptance of ADAS, their adoption is heavily influenced by the balance of perceived safety, perceived usefulness and perceived ease of use. In summary, older adults prefer Level 1 ADAS systems for their safety benefits without full automation. Perceived usefulness is crucial as these systems help address age-related driving limitations. While older adults value maintaining control and are less likely to adopt highly automated systems, they favor those providing warnings. Trust increases with experience, but false alerts and malfunctions can deter adoption. Barriers include high costs, lack of awareness, and complexity. Despite these challenges, interest in ADAS remains high, especially for features that reduce stress, and training programs or senior-friendly interfaces could improve acceptance. The gradual integration of these technologies, along with supportive measures such as user education and simplified interfaces, will be key to enhancing the comfort and trust of older adults in ADAS, ultimately leading to safer driving experiences. Therefore, designing user interfaces that are simple and user-friendly, particularly for senior drivers, is essential. Training and education programs tailored to older adults can further enhance understanding and comfort with ADAS, promoting safer and more confident use of these systems. The key limitations of this study include the potential exclusion of relevant studies, restriction to English-language research, and limited exploration of gender differences in ADAS perception among older drivers, highlighting the need for future research.

## Figures and Tables

**Table 1:** An overview of the six levels of ADAS.

Level	Automation Level	Typical Features
**Level 0**	No Automation	The human driver is responsible for all driving tasks.
**Level 1**	Driver Assistance	The vehicle features a single automated system (e.g., like Lane Keeping Assist (LKA), Adaptive Cruise Control (ACC), and Blind Spot Monitoring (BSM).
**Level 2**	Partial Automation	Systems can control both steering and acceleration/deceleration (e.g., Proactive Braking Intervention).
**Level 3**	Conditional Automation	The vehicle can perform most driving tasks under certain conditions but requires human intervention.
**Level 4**	High Automation	The vehicle is capable of performing all driving tasks in specific scenarios without human intervention.
**Level 5**	Full Automation	The vehicle can operate without human intervention in all scenarios.

**Table 2:** Overview of the studies.

First Authors	Year	Country	Methods	Level of ADAS	Construct	Main Results
[32]	2022	USA	Survey	1	Perceived safety, trust.	Older adults were more likely to adopt LKSs, BUCs, and ACC, as these technologies enhance driving control and safety. They were less likely to adopt an ABS due to concerns about its invasiveness.
[49]	2018	France	Focus group based on collective questionnaire sessions	1, 2, 3	Perceived usefulness	Although older adults had a positive view of ADAS and are open to adopt new technologies, they preferred systems that provide information or warnings rather than those that fully automate driving functions.
[45]	2015	Australia	Interview and survey	2	Perceived safety	Older drivers perceived ADAS as a primary factor influencing vehicle safety. They were more interested in being reassured about the effectiveness of ADAS in ensuring their safety rather than in understanding the technical details of how they work. They were open to adopting ADAS but perceived them as supplementary safety measures rather than substitutes for driving skills.
[37]	2016	USA	Experimental study (naturalistic driving guide and interviews)	1, 2	Perceived Usefulness	Older drivers expressed a high level of acceptance of ADAS, but with a lower incidence of use of LKA.
[38]	2018	Germany	Experimental study (with simulator, questionnaire, and interviews)	3	Trust, perceived usefulness	Older adults showed some acceptance of ADAS but have many concerns regarding safety. They preferred a more defensive driving style and desired to maintain control over the vehicle. They found ADAS useful for increasing their autonomy.
[46]	2017	Canada	Interview	1,2	Perceived usefulness, perception of safety, ease of use	Participants expressed a high level of usefulness, perception of safety, ease of use in ADAS.
[39]	2015	Germany	Experimental study (with simulator and questionnaire)	3	Trust	After the drive in the simulator, elderly participants showed higher trust.
[40]	2021	Germany	Experimental study (with simulator and questionnaire)	1	Perceived usefulness and trust	Older adults showed higher acceptance of ADAS, significantly influenced by perceived usefulness and trust.
[41],	2019	Japan	Experimental study (driving simulator and questionnaire)	2	Trust and acceptability	After the use of the simulator with ADAS, trust and acceptability of ADAS was improved.
[42]	2018	Japan	Experimental study (driving simulator and questionnaire)	2	Trust	Information sharing that includes visual content is effective in improving elderly drivers’ understanding of benefits and trust of the system.
[43]	2018	Japan	Experimental study (with simulator and questionnaire)	2	Trust	While many elderly drivers accepted proactive braking intervention, more than half did not accept proactive steering intervention.
[33]	2021	Japan	Survey	2	Perceived usefulness, ease of use	The results showed that social cohesion was a significant predictor of interest, usefulness, and ease of use of the ADAS’s acceptance.
[23]	2019	USA	Experimental study (naturalistic driving) with focus group	1, 2	Trust	Older drivers recognized the safety benefits of ADAS systems.
[47]	2018	USA	Interviews, focus group, and questionnaire	1, 2	Perceived usefulness, trust, perception of safety, perceived ease of use	Older adults believed that the use of ADAS could significantly reduce the stress associated with driving, improve efficiency and comfort during daily commutes, and save time. Despite recognizing the potential benefits of ADAS, many older adults highlighted a lack of trust in the technology itself.
[23]	2021	USA	Survey	1, 2	Usefulness, perceived safety, perceived ease of use	The study found that perceived usefulness and perceived safety are the most significant factors influencing older adults’ intention to use ADAS.
[35]	2023	Germany	Survey	2	Perceived usefulness	Perceived usefulness tended to increase with age.
[50]	2021	Japan	Experimental study (naturalistic driving) with telemetric data	1, 2	Risk perception	Elderly drivers exhibited a higher perception of road risk and tended to take additional safety measures, even when supported by ADAS.
[48]	2018	Canada	Interviews	2	Trust	Older drivers engaged in trust-building activities to understand the usability of this technology.
[36]	2023	USA	Survey	1, 2, 3	Perceived usefulness	Participants perceived the potential usefulness of ADAS technologies to support their driving.
[44]	2021	USA	Experimental study (with simulator and questionnaire)	2	Trust	ADAS training can improve female older adults’ trust and knowledge of ADAS.

## Data Availability

Not applicable.
